# Analysis of extracellular vesicles generated from monocytes under conditions of lytic cell death

**DOI:** 10.1038/s41598-019-44021-9

**Published:** 2019-05-17

**Authors:** Amy A. Baxter, Thanh Kha Phan, Eric Hanssen, Michael Liem, Mark D. Hulett, Suresh Mathivanan, Ivan K. H. Poon

**Affiliations:** 1 Department of Biochemistry and Genetics, La Trobe Institute for Molecular Science, La Trobe University, Melbourne, Victoria 3086 Australia; 2 Advanced Microscopy Facility, Bio21 Molecular Science and Biotechnology Institute, the University of Melbourne, Melbourne, Victoria 3010 Australia

**Keywords:** Cell death, Mechanisms of disease, Cell biology

## Abstract

Extracellular vesicles (EVs) are an important class of membrane-bound structures that have been widely investigated for their roles in intercellular communication in the contexts of tumor progression, vascular function, immunity and regenerative medicine. Much of the current knowledge on the functions of EVs pertains to those derived from viable cells (e.g. exosomes and microvesicles) or apoptotic cells (e.g. apoptotic bodies) whilst the generation of EVs from dying cells under non-apoptotic conditions remains poorly characterized. Herein, the release of EVs from THP-1 monocytes under conditions of primary necrosis, secondary necrosis and pyroptosis, was investigated. A comprehensive analysis of THP-1-derived EVs revealed that cells undergoing lytic forms of cell death generated a high number of EVs compared with viable or apoptotic cells *in vitro*. Differential centrifugation via 16,000 *g* and 100,000 *g* revealed that dying THP-1 cells release both medium and small EVs, respectively, consistent with the known characteristics of microvesicles and/or exosomes. In addition, large EVs isolated via 2000 *g* centrifugation were also present in all samples. These findings suggest that lytic cell death under both sterile and non-sterile inflammatory conditions induces monocytes to generate EVs, which could potentially act as mediators of cell-to-cell communication.

## Introduction

Cells that undergo physical trauma, infection or exposure to toxins can succumb to cell death via the downstream event of plasma membrane lysis, during which time intracellular contents can leak from the cells and osmotic homeostasis is lost. Such an occurrence is often accompanied by inflammation as damage associated molecular patterns (DAMPs) and/or pathogen-associated molecular patterns (PAMPs) are released, warning surrounding cells of tissue damage and triggering immune cell infiltration^1–3^. Depending on the mode of cell death, release of inflammatory and other cellular components may occur as an unregulated process resulting from plasma membrane damage, as in primary necrosis, or it may occur via the regulated formation of membrane pores, such as in the programmed cell death pathway of pyroptosis^4^. In additional to soluble intracellular contents, whether cells undergoing lytic forms of cell death can also release membrane-bound vesicles (also referred to as extracellular vesicles, or EVs) is not well understood.

EVs are typically classed into three main categories based on biogenesis and size, which include exosomes, generated via the endocytic pathway (30–150 nm), shed microvesicles, generated via budding from the plasma membrane (0.1–1 μm) and apoptotic bodies (1–5 μm), released from cells during the programmed cell death pathway of apoptosis^5–7^. Due to noteable overlap in size of EVs across these subcategories, considerable effort has been made within the EV field to establish standardized guidelines to accurately separate EVs into distinct subtypes, such as through detailed protein characterization of EV isolates^8^. Where appropriate, the use of alternative nomenclature to distinguish EVs based on physical characteristics such as size, density or other measures is increasingly adopted^5,8,9^. Although much attention has been paid to the characterization and functional significance of EVs derived from viable cells or apoptotic cells, less has been elucidated regarding the release of EVs from dying cells of non-apoptotic origin in which plasma membrane integrity is lost. Within this study we performed an in-depth characterization of EVs released by THP-1 monocytes following membrane lytic cell death via models of primary necrosis, secondary necrosis and pyroptosis^4,10,11^ which, despite their differing upstream mediators, converge at the downstream endpoint of membrane rupture and the release of intracellular contents^2,12^.

Primary necrosis is the result of cell death following exposure to extreme conditions or stimuli, resulting in non-specific permeability and/or rupture of the plasma membrane^13^. Primary necrosis of cells or tissue can occur in various settings including physical trauma, toxins or extreme temperatures^13^. In contrast, secondary necrosis is defined by the permeabilization of the plasma membrane following apoptosis, in which leakage of DAMPs such as nuclear protein HMGB1 can occur, thereby activating inflammatory responses^14^. Secondary necrosis occurs in the absence of sufficient apoptotic cell clearance and has been described in disease settings such as systemic lupus erythematosus^15^ and in solid tumors^16^. It should be noted that, although DFNA5 (also known as Gasdermin E) has been previously reported as a novel regulator of membrane lysis during secondary necrosis^17^, more recent findings suggest that, at least in THP-1 monocytes, Jurkat T cells and primary macrophages, this is not the case^18,19^. In addition to primary and secondary necrosis, cell death resulting in membrane permeabilization may also occur following inflammasome activation, such as via activation of the Nod-like receptor Protein 3 (NLRP3) inflammasome pathway, leading to pyroptosis^20^. Although several pathways to pyroptosis are known^21–23^, activation of the well-characterized NLRP3 pathway occurs in macrophages and monocytes following a variety of pathogenic and environmental stimuli and involves the upregulation of NLRP3 and pro-inflammatory cytokines IL-1β and IL-18 in their immature forms via TLR4 activation (e.g. by LPS), followed by inflammasome assembly mediated by microbial or environmental stimuli (e.g. the bacterial toxin, nigericin). During this process, cleavage of pro-caspase 1 into its active form enables subsequent cleavage of IL-1β and IL-18 into their mature forms. Simulatenously, Gasdermin D is cleaved by caspase 1 to enable N-terminal translocation to the plasma membrane, mediating membrane lytic cell death and the release of pro-inflammatory cytokines^24^.

To investigate the release of EVs by cells undergoing lytic forms of cell death in the current study, THP-1 monocytes were employed as a model cell line. Monocytes are a major class of myeloid immune cells that play a pivotal role in host immunity^25^. Upon activation, monocytes undergo differentiation either into macrophages, enabling the phagocytic removal of damaged, dead and cancerous cells^25^, or dendritic cells, which could mediate activation of adaptive immune responses via antigen presentation and subsequent T cell responses^26^. Concomitant to their role in immune surveillance, monocytes in circulation are exposed to a wide range of potential threats including infection by foreign pathogens^27^, accumulation at sites of necrotic tissue injury^28^ and autoimmunity-driven cytotoxicity^29^. Such conditions have the potential to induce necrotic phenotypes in monocytes, thereby activating immune responses via the release of DAMPs and/or PAMPs. Notably, the release of EVs by THP-1 monocytic cells under the necrotic conditions tested within this study have not been widely reported.

As described herein, compared with EVs released from apoptotic cells, lytic forms of cell death including primary necrosis, secondary necrosis and pyroptosis resulted in the generation of a markedly higher number of THP-1-derived EVs, consistent with the characteristics of microvesicles and/or exosomes. These findings highlight the need for further functional studies in which a potential role for these EVs in cell-to-cell communication, dead cell clearance or otherwise, may be elucidated.

## Methods

### Cell lines and cell culture

THP-1 monocytes were purchased from ATCC and cultured in RPMI-1640 supplemented with 5% fetal bovine serum (FBS) containing penicillin (50 U/ml and streptomycin (50 U/ml) (Life Technologies, Carlsbad, CA) and 0.2% (vol/vol) MycoZap (Lonza, Basel, Switzerland). All experiments involving EV isolation were performed in FBS-free RPMI-1640 supplemented with 1x insulin-transferrin-selenium (ITS) basal media supplement (Thermo Fisher, Waltham, MA).

### Cell death induction

Unless stated otherwise, THP-1 cells were seeded at a concentration of 1 × 10^6^/ml in ITS-RPMI media and were subjected to the four cell death stimuli as follows: To induce primary necrosis, cells were incubated at 37 °C for 3.25 h followed by incubation at 56 °C for 45 min (total incubation time 4 h); To induce secondary necrosis, cells were UV irradiated (150 mJ/cm^2^) using Stratagene UV Stratalinker 1800 (Agilent Technologies, CA) followed by incubation at 37 °C for 24 h; To induce pyroptosis, cells were primed with 1 μg/ml LPS (InVivogen, San Diego, CA) and incubated at 37 °C for 3 h. Cells were then treated with 10 μM nigericin (Sigma-Aldrich, St Louis, MO) for an additional 1 h (total incubation time 4 h); To induce apoptosis, cells were UV irradiated (150 mJ/cm^2^) using Stratagene UV Stratalinker 1800 followed by incubation at 37 °C for 4 h. Untreated control samples were incubated for either 4 h or 24 h at 37 °C. In all experiments involving UV irradiation, plastic covers of culture vessels were removed prior to irradiation to expose cells. In certain experiments, cells were pre-treated for 1 h with 50 μM Q-VD-OPh (Q-VD) or Ac-YVAD-cmk (YVAD) (Sigma-Aldrich).

### Flow cytometry assays

Samples were stained with annexin A5-FITC and TO-PRO-3 in annexin A5 binding buffer (Thermo Fisher) for 10 min at room temperature and incubated on ice prior to analysis. Flow cytometry-based cell death assays were performed on a BD FACSCanto II Flow Cytometer and BD FACSDiva software v6.1.1 (BD Biosciences, St Jose, CA) and subsequently analysed using FlowJo v10.5.3 software (Tree Star, Ashland, OR). Separation of samples into necrotic, viable and apoptotic groups was performed using the ‘two-stain’ gating strategy as previously described^30^.

### EV isolation

THP-1 cells in suspension were harvested and counted using a haemocytometer followed by centrifugation at 300 *g* for 5 min and resuspension at 1 × 10^6^ cells/ml in ITS-RPMI. Following treatment with cell death stimuli, differential centrifugation was performed utilizing a modified version of a previously published protocol by Kowal and colleagues^5^. Briefly, cells were centrifuged at 300 *g* for 10 min to remove whole cells. In some experiments, cell-free supernatants were collected for analysis following centrifugation at 2000 *g* (2 k) for 20 min to remove large EVs. In some experiments, sequential centrifugation was performed in which 2 k pellets were collected for analysis, followed by supernatants being centrifuged at 16,000 *g* (16 k) for 40 min and collected for analysis, followed by remaining supernatant then centrifuged in a at 100,000 *g* (100 k) for 60 min. EVs obtained from 2 k, 16 k and 100 k pellets were resuspended in 1 × PBS. 300 *g*, 2000 *g* and 16,000 *g* centrifugation was performed on an Eppendorf Centrifuge 5415 R (Eppendorf, Hamburg, Germany). 100,000 *g* centrifugation was performed on an Optima^TM^ Max-MP Ultracentrifuge (Beckman Coulter, Brea, CA).

### Nanosight tracking analysis (NTA)

Isolated EVs were prepared for NTA particle analysis on NS300^31^ (Malvern Panalytical, Malvern, UK) either by analysing supernatants, or by diluting pellets obtained via differential centrifugation in 1 × PBS until optimal particle concentration was obtained (determined as between 10–100 particles per frame, detection threshold level 3). Three × 60 sec measurements were captured per sample.

### Cryo electron microscopy

THP-1 (5 × 10^6^) monocytes per condition were suspended in RPMI-ITS growth media and subjected to cell death stimuli as described above. EVs isolated from 16 k and 100 k pellets via centrifugation as described above were then plunge frozen in liquid ethane and observed using a FEI Tecnai F30 at 200 kV with a defocus of ~ −5 micrometers. Micrographs were taken using a FEI CETA 4 k × 4 k camera with a dose of ~1,500 electrons/nm^2^.

### Protein quantification and immunoblot analysis

EVs isolated from THP-1 cells (as described above) along with whole cell pellets were lysed with Cytobuster (Merck, Kenilworth, NJ). Protein concentration was determined by Sypro® Ruby stain (Sigma Aldrich) as per manufacturer’s instructions, in the presence of Benchmark Unstained Protein Ladder (Life Technologies). Densitometry analysis was then performed using ImageJ software. Equal amounts of protein were separated via SDS-PAGE in the presence of SeeBlue Plus2 Pre-stained Protein Standard (Thermo Fisher) followed by western transfer onto PVDF membrane. Membrane was blocked with 5% skim milk powder in 1 × PBS followed by overnight incubation with the primary antibodies to Alix [3A9] (Cell Signaling Technology, Danvers, MA)^32^, ARF6 [ab77581] (Abcam, Cambridge, UK)^33^, CD81 [M38] (Life technologies)^34^ and Calreticulin [ab22683] (Abcam)^35^ in 1% BSA in PBST at 4 °C, followed by three 10 min wash steps in PBST. Membranes were then incubated with HRP-conjugated sheep anti-mouse antibodies (1:5000, Millenium Science), Li-Cor goat-anti rabbit or goat anti-mouse IRDye 800CW (1:10000, Millennium Science) in 1% BSA in PBST for 1 h at RT, followed by washing as described for primary antibodies. HRP signal was developed using ECL (GE Lifesciences, Boston, MA) and imaged using the Syngene G:Box gel documentation and analysis system (Syngene, Bangalore, India). IRDye signal was imaged using the LiCOR Odyssey infrared scanner (Millenium Science).

### LDH cell lysis assay

The release of lactate dehydrogenase (LDH) from permeabilized cells was measured using the LDH Cytotoxicity Assay Kit II (Abcam), according to the manufacturer’s instructions. Briefly, 5 × 10^4^–1 × 10^5^ cells were seeded into clear 96-well tissue culture plates and induced to undergo cell death via the four stimuli as described above. Culture supernatants were incubated with LDH reaction mix for 0.5–1 h and absorbance at 450 nm was measured using SpectraMax M5e Plate Reader (Molecular Devices, Sunnyvale, CA) and analyzed using the SoftMaxPro 5.2 software (Molecular Devices). Cell lysis was then calculated as a percentage of total lysis as determined by 30 min incubation with LDH cell lysis buffer.

### Confocal laser scanning microscopy (CLSM)

THP-1 cells or isolated EVs were adhered to 4- or 8-well live cell imaging Nunc™ Lab-Tek™ II chamber slides (Nunc, Rochester, NY) with 1% Poly-L-Lysine and imaged on a Zeiss LSM 780 or Zeiss LSM 800 confocal microscope (Zeiss, Oberkochen, Germany) using a 63x oil immersion objective. Microscope chamber was heated to 37 °C with 5% CO_2_. In certain experiments, imaging was performed in the presence of 1 μg/ml propidium iodide (PI). Images were then analysed using Zen software (Zeiss).

### Dynamic light scattering (DLS)

DLS experiments were performed in indicator-free RPMI containing 1 × ITS supplement. Following cell death induction, 2 k pellets were resuspended in 1 × PBS and analysed using a Zetasizer Nano ZS instrument (Malvern Panalytical)^36^. Experiments were performed in triplicate, using the non-negative least squares method after the polydispersity index of the cumulant fit was confirmed to be less than 0.7. All experiments were performed at 25 °C.

## Results

### THP-1 monocytes undergoing membrane-lytic cell death release EVs

**Figure 1:**
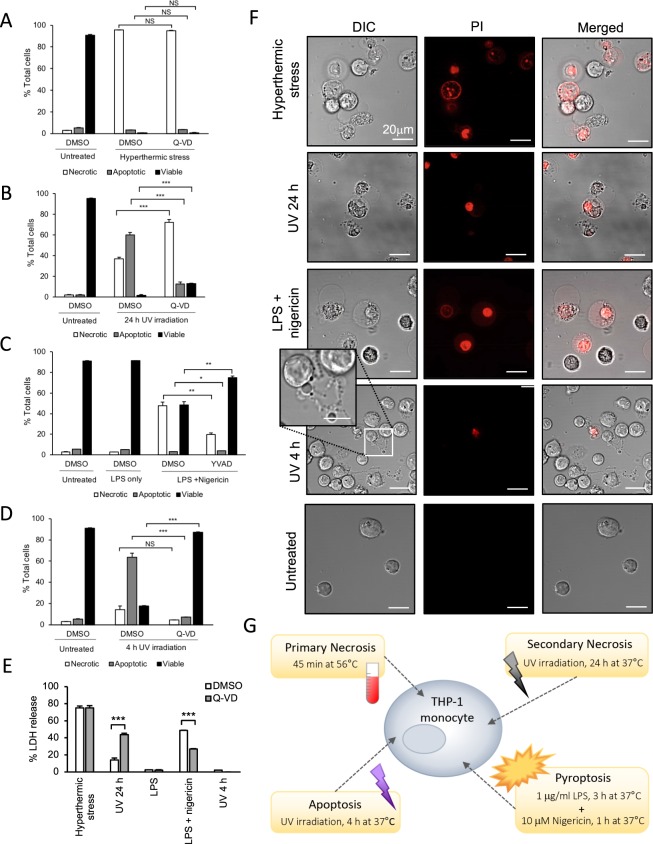
THP-1 monocytes undergo membrane lysis under conditions of primary necrotic, secondary necrotic and pyroptotic cell death. THP-1 cells subjected to (**A**) hyperthermic stress, (**B**) 24 h UV irradiation, (**C**) LPS/nigericin treatment or (**D**) 4 h UV irradiation, pre-treated with caspase inhibitors Q-VD (**A**,**B**,**D**) or YVAD (**C**) or DMSO (vehicle control), analysed by flow cytometry to determine relative numbers of membrane permeabilized (necrotic), apoptotic and viable populations. (**E**) Membrane lysis of THP-1 cells as demonstrated via the LDH cytotoxicity assay in the presence or absence of Q-VD for each of the four cell death treatments as described in (**A–D**). (**F**) Representative confocal laser scanning microscopy (CLSM) micrographs of THP-1 cells imaged in the presence of PI under the four treatment conditions as described in (**A–D**). Left panel = differential interference contrast (DIC); middle panel = PI; right panel = merged. Enlarged panel (DIC, UV 4 h) highlights string-like membrane protrusions generated by apoptotic THP-1 cells. (**G**) Schematic illustrating the four cell death models. Data are representative of at least two independent experiments. In (**A–E**), unpaired, two-tailed student t-tests were performed, NS = not significant, *P < 0.05, **P < 0.01, ***P < 0.001. Error bars = S.E.M. (N = 3). Scale bars in (**F**) represent 20 μm; scale bar in enlarged panel = 10 μm.

Firstly, to confirm the fate of THP-1 monocytic cells exposed to different cell death stimuli and the role of caspases in cell death induction, the following four cell death models were established: (i) hyperthermic stress (primary necrosis model); (ii) UV irradiation, 24 h incubation (secondary necrosis model); (iii) LPS/nigericin treatment (pyroptosis model), and (iv) UV irradiation, 4 h incubation (apoptosis model). The proportion of viable, apoptotic, and membrane permeabilized (necrotic) THP-1 cells was determined via flow cytometry, using an annexin A5 (binds phosphatidylserine on dying cells) and TO-PRO-3 (DNA binding dye stains viable, apoptotic and necrotic cells differentially) based on an approach previously described by Jiang and colleagues^30^. Detailed gating strategy and FACS dot plots are included as Supplementary Figures S1–S2. Hyperthermically stressed cells underwent substantial membrane permeabilization (96%), which was unaffected by pan-caspase inhibitor Q-VD treatment. Notably, hyperthermic stress-treated cells underwent minimal apoptosis (Fig. 1A). UV-treated cells incubated for 24 h also underwent membrane permeabilization (37%), whilst 60% still appeared apoptotic (Fig. 1B). Q-VD treatment reduced the amount of apoptotic cells by 47%, suggesting effective caspase 3/7 inhibition of cells undergoing apoptosis. Interestingly, in addition to an expected corresponding increase in viable cells in the Q-VD-treated samples, a significant increase in membrane-permeabilized cells compared with DMSO-treated cells, was also observed (Fig. 1B). This observation could indicate that the effect of caspase inhibition on UV 24 h treated THP-1 cells may trigger an alternative lytic cell death pathway. 48% of LPS/nigericin-treated cells underwent membrane permeabilization, which was reduced to less than 20% by the caspase-1 specific inhibitor, YVAD treatment (Fig. 1C). This decrease in membrane permeabilized cells corresponded with a 27% increase in viable cells, suggesting pyroptosis was successfully induced. Although an increase in the population of apoptotic cells in the DMSO versus YVAD-treated samples was statistically significant, the total levels of apoptosis in these two groups were both under 4%. It should be noted that comparable inhibition of cell death in THP-1 cells subjected to LPS/nigericin treatment was also observed in the presence of the pan-caspase inhibitor, Q-VD (data not shown). Conversely to these three models, cells incubated for 4 h following UV irradiation (apoptosis model) demonstrated minimal membrane permeabilization but a high proportion of apoptotic cells (64%), reduced to 7% by Q-VD pre-treatment. Q-VD-mediated inhibition of apoptosis also corresponded with a 70% increase in viable cells (Fig. 1D).

To further demonstrate the degree of membrane permeabilization following cell death induction via the four described models, the LDH release assay, in which cell lysis is determined by the release of the membrane impermeable 140 kDa cytosolic protein, LDH into the extracellular medium, was employed. While hyperthermic stress and LPS/nigericin treatments induced comparable levels of membrane permeabilization to those observed via flow cytometry, a decrease in cell lysis was seen in UV-treated cells incubated for 24 h (Fig. 1E). As observed in Fig. 1B, the presence of pan-caspase inhibitor Q-VD also promoted cell lysis  in UV-treated cells incubated for 24 h. Consistent with the observed YVAD-mediated inhibition of pyroptotic cell death in Fig. 1C, inhibition of cell lysis in LPS/nigericin-treated samples was also seen in Q-VD-treated samples (Fig. 1E). The morphology of THP-1 cells subjected to cell death induction via the four models as described above was also monitored via CLSM (Fig. 1F). Hyperthermic stress-, UV 24 h- and LPS/nigericin-treated cells demonstrated typical ‘necrotic’ phenotypes including swelling, large stationary membrane blebs and uptake of the membrane impermeable DNA binding dye propidium iodide (PI)^37^. In comparison, THP-1 cells following UV 4 h treatment displayed characteristics typical of monocytes undergoing apoptosis, including the formation of apoptotic bodies and string-like membrane protrusions known as beaded apoptopodia^38^ in the absence of large plasma membrane blebs and with minimal PI uptake (Fig. 1F). Together, these data validate the four different models of cell death induction, with three of these models able to induce lytic cell death in THP-1 monocytes (shown as schematic in Fig. 1G).

**Figure 2:**
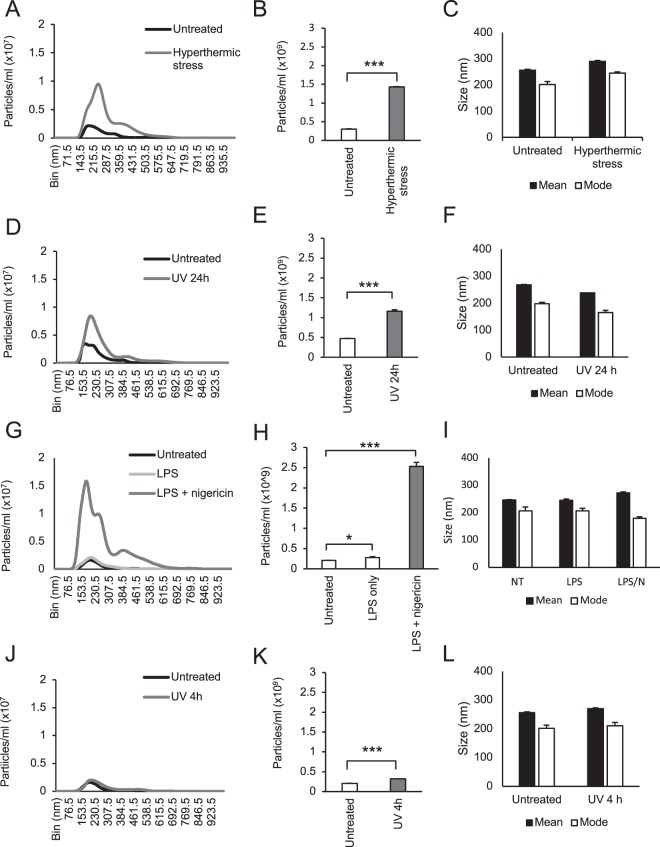
THP-1 monocytes release EVs following treatment with primary necrotic, secondary necrotic, pyroptotic and apoptotic stimuli. NTA analysis of cell-free supernatants isolated following 300 *g* and subsequent 2000 *g* centrifugation, collected from THP-1 cells following hyperthermic stress (**A–C**), 24 h UV irradiation (**D–F**), LPS + nigericin treatment (or LPS alone) (**G–I**) or 4 h UV irradiation (**J–L**), compared with supernatants from untreated control THP-1 cells. Histograms in (**A**,**D**,**G**,**J**) represent particle concentration vs size distribution. Bar graphs in (**B**,**E**,**H**,**K**) illustrate mean particles concentrations for each of the conditions. Mean and mode EV size for each of the conditions are presented as bar graphs in (**C**,**F**,**I**,**L**). Data are representative of three independent experiments. Unpaired, two-tailed student t-tests were performed, *P < 0.05, ***P < 0.001. Error bars = S.E.M. (N = 3).

Next, to determine whether THP-1 cells release EVs during membrane-lytic forms of cell death, cells were subjected to the four cell death stimuli, followed by analysis of cell-free supernatants via NTA, which enables quantitative measurement of particle size and concentration in solution^31^. An increase in particle concentration was observed following all four cell death treatments above the levels of untreated controls, as demonstrated via size distribution of particles (Fig. 2A,D,G,J) and quantification of vesicle concentration (Fig. 2B,E,H,K). LPS/nigericin treatment induced the greatest increase in particle concentration, whilst the UV 4 h treated sample displayed a comparatively modest increase (Fig. 2H,K). It should be noted that, although LPS treatment alone induced a modest particle concentration increase, the addition of nigericin increased this by approximately 9-fold, indicating that induction of pyroptosis specifically, was predominantly responsible for EV generation under this condition (Fig. 2H). Mean particle sizes from cell-free supernatants ranged between 200–300 nm across samples. A decrease in mode size of particles was also observed across all conditions measured, compared with mean values (Fig. 2C,F,I,L). These data suggest that, whilst the total range of EV sizes included those that were much larger e.g. greater than 500 nm, the majority of EVs were much smaller, i.e. below 250 nm. Based on these observations, it can be inferred that THP-1 monocytic cells subjected to membrane-lytic cell death release high quantities of particles that are consistent with the known sizes of EVs including microvesicles (0.1–1 μm) and/or exosomes (30–150 nm).

### EV release by heat-stressed, UV-irradiated and LPS/nigericin treated cells occurs at the onset of membrane lysis, in a time-dependent manner

**Figure 3:**
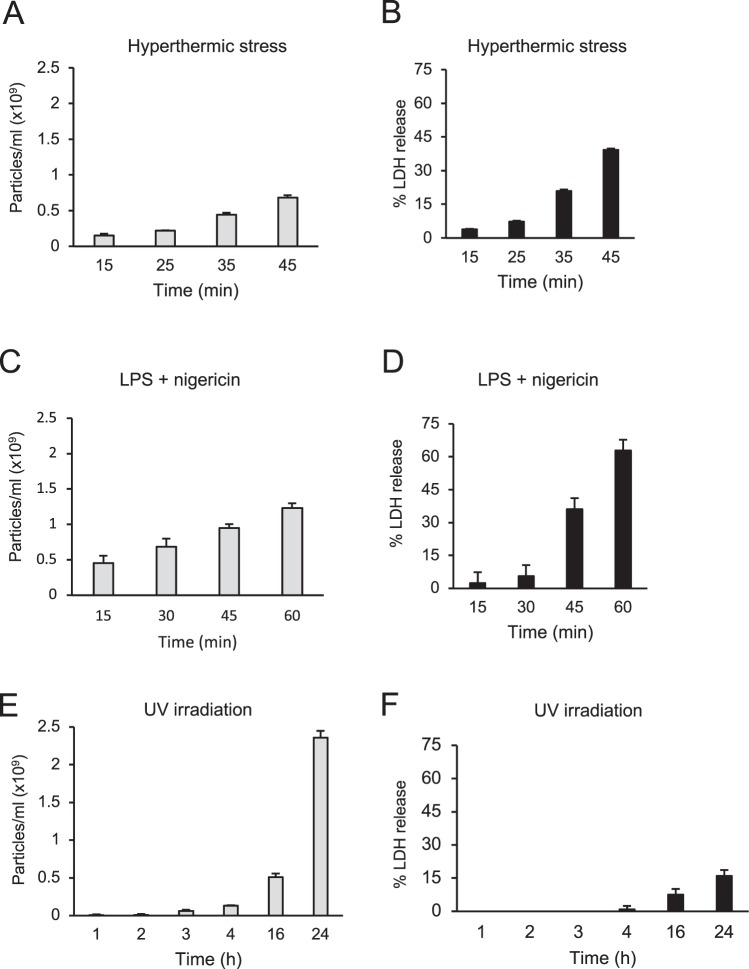
EV release by THP-1 cells undergoing primary necrosis, secondary necrosis and pyroptosis occurs simultaneously to membrane permeabilization. NTA analysis of supernatants of THP-1 cells subjected to (**A**) hyperthermic stress, (**C**) LPS/nigericin or (**E**) UV irradiation, collected at intermittent time points spanning total treatment times. Supernatants were isolated for NTA analysis following 300 *g* and subsequent 2000 *g* centrifugation. Values displayed represent particle concentration above levels of untreated samples. Total membrane lysis as determined by LDH cytotoxicity assay was performed at corresponding time points for the same treatment conditions (**B**,**D**,**F**). Data are representative of three independent experiments. Error bars = S.E.M. (N = 3).

To gain insights into the kinetics of EV release by THP-1 cells relative to membrane permeabilization, cell-free supernatants from THP-1 cells were collected for analysis via NTA at intermittent timepoints spanning total treatment times, following cell death induction (Fig. 3A,C,E). LDH release assays were used to assess membrane permeabilization. (Fig. 3B,D,F). In both hyperthermic-stressed and LPS/nigericin-treated samples, an increase in EV concentration above basal levels from 15 min could be seen (Fig. 3A,C), corresponding to an increase in cell lysis at this time point, with increasing cell lysis and EV concentrations observed across subsequent timepoints of 25, 35 and 45 min for hyperthermic stress treatment (Fig. 3B) and 30, 45 and 60 min for LPS/nigericin treatment (Fig. 3D). Extended timepoints of 90 min and 180 min were performed for LPS/Nigericin samples, demonstrating a continuous increase in EV concentration corresponding with increased LDH release to maximum levels (Fig. S9). For UV-treated cells, although by 4 h a modest increase in EV concentration was observed in UV-treated samples (Fig. 3E), a marked increase in cell lysis was not seen over this time (Fig. 3F). However, EV concentration increased by the 16 h and 24 h timepoints along with an increase in cell lysis at these later timepoints (Fig. 3E,F). Together, these data demonstrate that during membrane-lytic cell death of THP-1 cells, EV release occurs at the onset (or preceding the onset) of membrane lysis.

### Differential centrifugation and immunoblot analysis reveal a heterogeneous population of THP-1-derived EVs across 2 k, 16 k and 100 k pellets

The removal of large EVs from cell-free supernatants is commonly employed by performing slow and medium-speed centrifugation, prior to high-speed centrifugation to isolate small EVs including exosomes and small microvesicles^39,40^. However, since dying cells under apoptotic conditions are known to release large EVs (e.g. apoptotic bodies) of functional significance^7^, and given that the release of EVs by cells undergoing lytic (non-apoptotic) cell death has been poorly characterized to date, we sought to determine whether larger EVs (i.e. those isolated by slower centrifugation at 2 k and 16 k *g*) are also released by cells undergoing primary necrosis, secondary necrosis and pyroptosis. Therefore, we next performed EV isolation via subsequent differential centrifugation steps at 2 k, 16 k and 100 k *g*, following removal of the whole cell pellet at 300 *g*.

**Figure 4:**
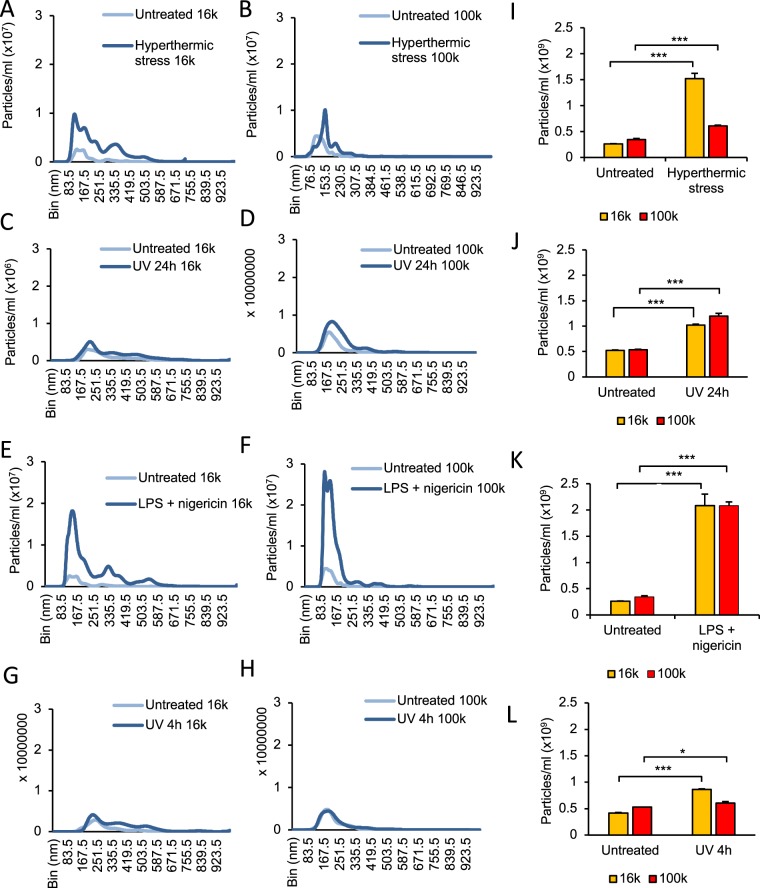
EVs released by THP-1 cells following cell death induction were detected via NTA in both 16 k and 100 k pellets. NTA analysis of 16 k (left) and 100 k (center) pellets of THP-1-derived EVs subjected to hyperthermic stress (**A**,**B**), UV 24 h (**C**,**D**), LPS/nigericin treatment (**E**,**F**) or UV 4 h (**G**,**H**). (**I–L**) Bar graphs illustrate mean particles/ml comparisons between 16 k and 100 k pellets from (**A–H**). Data are representative of three independent experiments. Unpaired, two-tailed student t-tests were performed, *P < 0.05, ***P < 0.001, NS = not significant. Error bars = S.E.M. (N = 3).

Initial attempts to analyse EVs isolated in the 2 k pellets via NTA generated noise detection warnings (data not shown). This occurrence was likely due to the presence of particles within these fractions being beyond the 2,000 nm upper detection limit of the instrument as defined by the manufacturers. We therefore performed size distribution analyses on 2 k pellets by DLS as described below. Size distribution of 16 k pellets (Fig. 4A,C,E,G) and 100 k pellets (Fig. 4B,D,F,H), and quantification of EV concentrations (Fig. 4I–L) were measured via NTA, revealing significant increases in EV concentrations compared to untreated controls for each of the hyperthermic stress-, UV 24 h- and LPS/nigericin-treated samples, across both 16 k and 100 k pellets samples (Fig. 4I–L). Notably, mean concentrations of EVs released by hyperthermically stressed and UV 4 h-treated cells were markedly lower in the 100 k pellet than in the 16 k pellet (Fig. 4I,L). Mean EV sizes of 100 k pellets (147 nm-247 nm) were consistent with typical size ranges of microvesicles (0.1–1 μm) and/or exosomes (30–150 nm), whilst mean EV sizes of 16 k pellets (189 nm-364 nm) were consistent with those of microvesicles.

**Figure 5:**
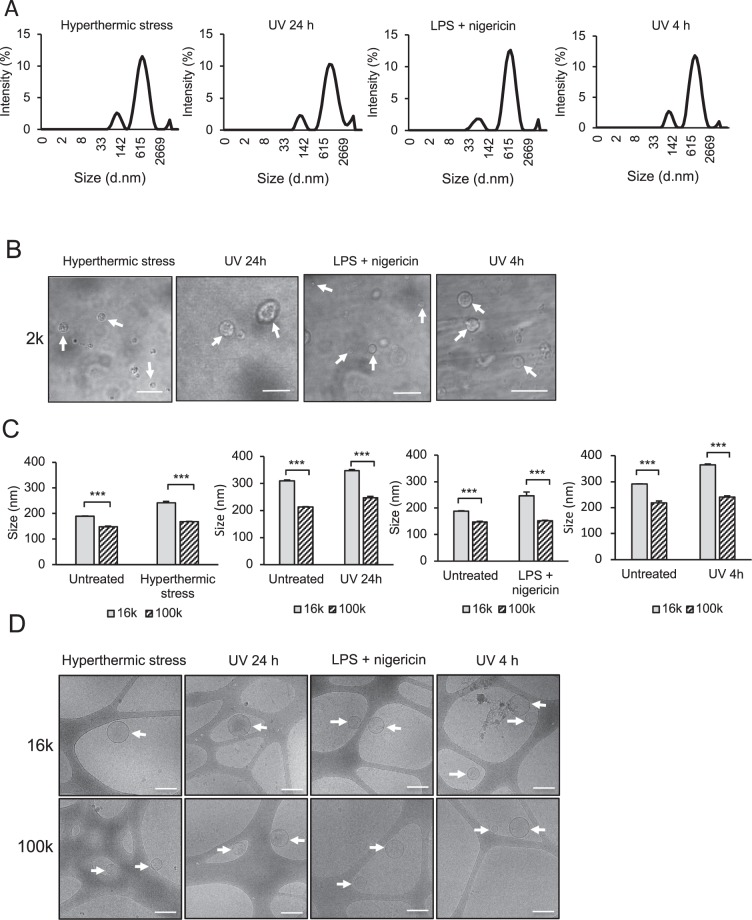
EVs released by THP-1 cells under conditions of membrane-lytic cell death are heterogeneous in size. (**A**) Representative size distribution by intensity histograms of EVs isolated from cell-free supernatants via 2 k centrifugation, as determined DLS, where x axis represents particle size distribution in nm (d.nm) and y axis represents intensity distribution of light scattering by particles in solution (Intensity %) (**B**) CLSM DIC images depict EVs isolated from 2 k pellets, indicated by white arrows. Scale bars = 10 μm. (**C**) NTA analysis of mean particle size of THP-1-derived EVs from 16 k and 100 k pellets. (**D**) CryoEM images depict EVs isolated following 16 k and 100 k centrifugation, indicated by white arrows. Scalebars = 200 nm. In (**C**), unpaired, two-tailed student t-tests were performed, ***P < 0.001. Error bars = S.E.M. (N = 3). Data in (**A–C**) are representative of three independent experiments.

2 k pellet analysis by DLS revealed the presence of EVs in each of the four cell death models (Fig. 5A, Supplementary Table 1). High polydispersity index (PdI) scores (>0.5) indicated a broad size distribution across samples, confirmed by the presence of multiple peaks ranging from submicron-sized (i.e. within the size ranges of microvesicles and/or exosomes), to larger (>4 μm) EVs (i.e. within the size range of apoptotic bodies or other large EVs, ~1–5 μm) (Supplementary Table 1). Consistent with these findings, CLSM analysis of isolated EVs from 2 k pellets also confirmed the presence of large, non-aggregated EVs within each sample (Fig. 5B). Although particle concentration could not be ascertained for 2 k pellets, we determined total protein yield of each of the 2 k, 16 k and 100 k pellets via SDS-PAGE (Fig. S3A,B) and performed subsequent densitometry analysis (Fig. S4A,B). We acknowledge that total protein yield is a limited quantification measure, since EVs derived under the different cell death conditions could contain highly variable cargoes and hence protein yields may not truly reflect differences in total EV numbers. Of particular interest, however, was the difference in protein yield between the UV 4 h (apoptotic) and the UV 24 h (secondary necrotic) pellets, as these could represent changes in levels and/or composition of EVs released by cells having transitioned from apoptotic to secondary necrotic states (Fig. S4A). To interrogate this notion, relative fold increases in total protein content in UV 24 h compared to UV 4 h pellets were determined. Interesting, whilst no increase was observed in the UV 24 h 2 k pellet above that of the UV 4 h 2 k pellet, an increase in protein concentration in both the 16 k and 100 k samples could be seen (Fig. S4B). These data suggest that whilst apoptotic bodies are released during the apoptotic phase of cell death, the levels of smaller EVs (i.e. those isolated via 16 k–100 k centrifugation) could increase once cells have entered the secondary necrotic phase. NTA analysis of 16 k and 100 k pellets revealed a decrease in mean EV size of those isolated from 100 k pellets compared to 16 k pellets (Fig. 5C). CryoEM was also performed on 16 k and 100 k pellets to confirm the presence of non-aggregated EVs in each of the hyperthermically stressed, UV-irradiated and LPS/nigericin-treated samples (Fig. 5D).

**Figure 6:**
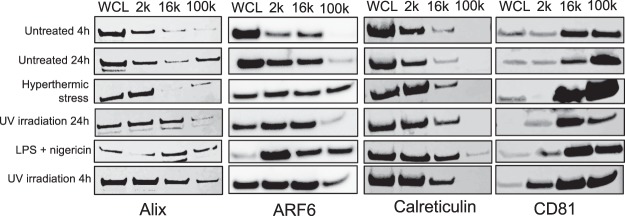
Protein profiles of EVs released by THP-1 cells under conditions of membrane-lytic cell death are characteristic of microvesicles and/or exosomes. (**E**) Equal amounts of protein from whole cell lysate (WCL) and 2 k, 16 k and 100 k pellets following hyperthermic stress, 24 h UV irradiation, LPS + nigericin or 4 h UV irradiation treatments or from untreated controls (4 h or 24 h), were analysed via western blotting. Individual membranes are derived from the same experiment and blots were processed in parallel. Cropped images are displayed, full-length blots are displayed in Supplementary Figs S5–S8.

Next, protein content-based EV characterization via immunoblot analysis was performed on isolated EVs, in which the presence of endosomal trafficking accessory protein, Alix (an exosome-specific marker), membrane trafficking protein, ARF6 (associated with plasma membrane microvesicle shedding^6^), the endoplasmic reticulum resident protein, calreticulin (expected to be least abundant in small EVs/100 k pellet) and the membrane tetraspanin CD81 (a commonly used marker for small EVs, expected to be most abundant in 100 k pellet fractions), was determined. While calreticulin was largely depleted in 100 k pellets and reduced in 16 k pellets in all samples, CD81 was enriched in 16 k and 100 k pellets compared to whole cell lysate (WCL) and 2 k pellets (Fig. 6). These findings are consistent with the expected distributions of such EV protein markers in EVs of these sizes^5,8,41^. Alix was detected across all cell-free fractions although notably decreased in both 16 k and 100 k pellets of hyperthermic stress-treated and untreated (4 h) samples (Fig. 6). ARF6 was also present in all EV fractions undergoing cell death, whilst being abundant in both 16 k and 100 k fractions of hyperthermic stress- and LPS/nigericin-treated samples (Fig. 6). Uncropped immunoblots are included as supplementary material (Figs S5–S8). These data demonstrate that cells undergoing membrane-lytic cell death release intact EVs that are heterogenous in size and, together with the distribution of known EV protein markers, reflect the characteristics of microvesicles and/or exosomes.

## Discussion

In the present study, the ability of THP-1 monocytes subjected to three separate models of membrane lytic cell death to release EVs was investigated. In addition to our findings supporting previous reports of EV generation from apoptotic cells, we have also demonstrated for the first time that cells undergoing membrane lytic cell death via hyperthermic stress, UV-induced secondary necrosis and pyroptosis can also generate a variety of EVs under *in vitro* conditions.

Kinetics analysis revealed that THP-1 cells undergoing hyperthermic stress treatment generated significantly elevated levels of EVs preceding the onset of membrane permeabilization, with increasing EV concentration detected simultaneously with cell lysis. Although the effects of hyperthermic stress on EV generation has been described previously for primary human monocytes, T cells, B cells and epithelial cells^39,42,43^, no previous studies on THP-1 monocytes under the conditions tested in the present study have been reported. The following are examples of previous reports in other cell types. Heat-shocked Jurkat T cells and Raji B cells (40 °C for 1 h) expressing the natural killer cell receptor NKG2D promoted the release of exosomes that possessed immunosuppressive function^39^, whilst the release of exosomes from MCF7 cells following incubation at 45 °C for 1 h was shown to induce apoptosis and inhibit proliferation in neighbouring cells^42^. Interestingly, in the latter study, although hyperthermic exposure induced a functional change in the exosomes, the overall EV concentration generated by heat-treated cells was not significantly greater than in untreated controls. In another study, exosome release by primary blood mononuclear cells (PBMCs) following exposure to 40–43 °C temperatures for 1 h induced an increase in HSP70 without increasing total exosomal concentration, compared with untreated controls^43^. These reports differ from our findings, in which an approximately 5-fold increase in particle concentration was detected within the supernatant in hyperthermically stressed THP-1 cells compared with untreated controls after 45 min, indicating that the greater temperature used within the present study (56 °C) may have a considerable impact on the degree of cellular stress and, hence EV release. It is also worth noting that, in these previous reports, cell death was either not reported^39,43^ or occurred via apoptosis rather than necrosis^42^, further suggesting that temperature variation and therefore potential cell death pathway induced, could alter both the amount and mode of EV generation. However, as no direct comparisons can be made between different cell types, further investigations into the release of EVs by hyperthermically stressed primary monocytes as well as other cell types would be necessary determine whether the observed effects are cell-type specific.

Interestingly, total protein yield of hyperthermically stressed THP-1 cells within the present study was markedly lower than in any other conditions tested, suggesting that EVs released by cells undergoing rapid primary necrosis may undergo more extensive membrane damage in which rapid membrane shedding, rather than the regulated packaging of cargo and secretion via the endocytic pathway, occurs. This notion is supported by our NTA analysis of 16 k vs 100 k pellets of hyperthermic stress-derived EVs, in which a higher proportion of EVs isolated via 16 k pellets than 100 k pellets, was detected. Our immunoblot analysis also supports this, as the depletion of Alix but presence of ARF6 in 16 k and 100 k EV fractions, was observed. Cells undergoing primary necrosis, either via hyperthermic stress as discussed above, or via other environmental or physical stimuli, can induce a sterile inflammatory response via the release of intracellular contents into the extracellular surroundings^2^. It would therefore be of future interest to determine the contents of EVs released by monocytes via hyperthermic stress-induced primary necrosis, and whether they bear any functional significance with respect to mediating inflammation.

A marked increase in EV concentration in secondary necrotic samples compared with untreated controls was also observed in the present study. Importantly, whilst only a modest increase in EV release was observed during the apoptotic phase of THP-1 cell death, particle concentration increased over time at both 16- and 24-hour time points. This coincided with modest but observable increases in cell lysis at these time points, suggesting that as cells begin to undergo permeabilization, EVs are released. In addition, the observation that only the levels of EVs isolated from 16 k–100 k pellets, but not those isolated via 2 k pellets increase when cells undergo secondary necrosis (above levels observed during apoptosis), supports the notion that the generation of apoptotic bodies is a highly regulated process, occurring within a specific time frame and ceasing when cells become necrotic^38,44^. This is, to our knowledge, the first instance in which secondary necrotic EVs have been characterized in this way. Whilst it is tempting to speculate that the smaller EVs generated during secondary necrosis could potentially act as mediators of cell-to-cell communication by dying cells, functional studies are clearly required. Of future interest would be to determine the presence of known cellular proteins released during secondary necrosis, such as HMGB1, within the observed EVs. Importantly, future investigations into whether EVs released by secondary necrotic cells could potentially serve as biomarkers for disease states, are also warranted.

Pyroptotic THP-1 monocytes released a high number of EVs upon LPS/nigericin treatment, compared with untreated control samples. A range of different sized EVs could be visualized via CLSM (2 k pellet) and via CryoEM (16 k/100 k pellet) and displayed protein characteristics indicative of microvesicles and possibly exosomes. A number of previous studies have examined the release of EVs by inflammasome-activated monocytes and macrophages. For example, Zhang and colleagues performed a proteomics analysis on exosomes/small EVs released by murine bone marrow-derived macrophages following LPS/nigericin-mediated inflammasome activation in which NFκB signalling was activated^45^. Likewise, exosomes/small EVs isolated from LPS-stimulated primary human monocytes induced caspase-1 mediated apoptosis in co-cultured vascular smooth muscle cells^40^. Various other upstream mediators of NLRP3 inflammasome activation such as ATP^46,47^ and β-glucan^48^, have also been reported to induce EV release, including a 2001 study by Mackenzie and colleagues who observed that IL-1β-containing shed microvesicles from LPS/bzATP treated THP-1 cells could mediate rapid IL-1 receptor activation in target Hela cells^47^. Importantly, in these previous studies, pyroptotic cell death in inflammasome-activated cells was either not measured, or shown not to occur. Therefore, whilst EV release by cells undergoing inflammasome activation can occur via various stimuli, the specific effect of pyroptotic cell death on EV release has not been previously reported. In the current study, LPS/nigericin-mediated cell lysis as a determinant of pyroptotic cell death temporally coincided with increased EV concentration, suggesting that, as observed in hyperthermically stressed and secondary necrotic THP-1 monocytes, pyroptotic EVs are generated during the cell death process. Pyroptotic cell death is an important host response mechanism to pathogenic infection, whereby infected cells undergo ‘suicide’, thereby limiting pathogen proliferation. Subsequently, the release of inflammatory cytokines can promote immune cell infiltration and stimulate adaptive immunity^49^. Our findings within the present study suggest that, in addition to the previously reported release of EVs by activated cells^40,47^, dying pyroptotic cells also release EVs. This observation prompts the question of whether the release of such EVs could contribute to host immunity through the transfer of inflammatory cytokines or other factors to distal cellular targets. Indeed, this concept has been proposed previously by Buzas and colleagues^50^, whilst a similar parallel can be drawn against observations made of bacterial outer membrane vesicles (OMVs), described as ‘hand grenades’ that, once released by dying bacteria can act as potent propagators of inflammation^51^. In the context of human monocytic EVs released during pyroptosis, the stability and cargo of these EVs remains to be determined. Further investigations regarding these and other questions, such as whether pyroptotic EVs also contain proinflammatory cytokines, caspase-1, ASC or other factors with the capability of propagating immune signalling, are clearly warranted.

### Concluding remarks

EVs continue to emerge as an important class of cell-derived structures that are capable of mediating profound effects on biological processes and pathways, under both physiological and pathological conditions. In this study, we have demonstrated via a comprehensive analysis of THP-1 monocytes, that these cells undergoing lytic forms of cell death can also release an abundance of EVs, a phenomenon previously unreported. Of interest in future studies would be to investigate the release of EVs by primary monocytes and indeed other cell types. Comparisons between EVs of different cell types/tissues of origin could reveal differences in both the yield and composition of EVs under the conditions tested and hence provide additional insights into the nature of EV generation by dying cells. With this in mind, the cargo that dying cell-derived EVs release, the function they serve and whether such EVs can be exploited as therapeutic targets for disease, remain to be elucidated and will be important ongoing questions for further investigation.

## Supplementary information

Supplementary Information
