# Supplementary material for: Analysis of extracellular vesicles generated from monocytes under conditions of lytic cell death
Source: Sci Rep. 2019 May 17;9:7538. doi: 10.1038/s41598-019-44021-9 (PMC6525174; doi:10.1038/s41598-019-44021-9)
Supplement: Supplementary file 1 — Supplementary Information [file 41598_2019_44021_MOESM1_ESM.pdf]

Analysis of extracellular vesicles generated from monocytes under conditions of lytic cell death: Supplementary Information

[1]Amy A Baxter, [1]Thanh Kha Phan, [2]Eric Hanssen, [1]Michael Liem, [1]Mark D Hulett, [1]Suresh Mathivanan, [1]Ivan KH Poon.

[1]Department of Biochemistry and Genetics, La Trobe Institute for Molecular Science, La Trobe University, Melbourne, Victoria 3086, Australia.

[2]Advanced Microscopy Facility, Bio21 Molecular Science and Biotechnology Institute, the University of Melbourne, Melbourne, Victoria 3010, Australia

Correspondence should be addressed to Amy A Baxter or Ivan KH Poon
Email: a.baxter@latrobe.edu.au, i.poon@latrobe.edu.au
Tel: +61 3 9479 2211
Address: Department of Biochemistry and Genetics, La Trobe Institute for Molecular Science, La Trobe University, Melbourne, Victoria 3086, Australia.
Key words: Extracellular vesicles, necrosis, pyroptosis

Supplementary Figure legends:

Supplementary Table 1. DLS modelling data. Peak parameters derived from Non-Negative Least Squares fitting of the autocorrelation function for each of the four cell death conditions. Cumulant polydispersity indices (PdI) are also shown. Data are representative of three independent experiments.

Figure S1. Gating strategy and representative FACS dot plots utilised in Figure 1 A-B to assess cell death in THP-1 cells. Cells double-stained with TO-PRO-3 and A5-FITC were gated in four stages to isolate 'viable', 'apoptotic' and 'necrotic' (membrane permeabilized) cells, based on a modified version of method previously described by Jiang and colleagues (see Methods section): Non-cellular particles/ debris as defined by small forward and side scatter were removed from analysis (Far left column); TO-PRO-3^{high}/A5^{high} cells were defined as 'Necrotic' (middle left); A5^{high} cells from non- TO-PRO-3^{high}/A5^{high} population were defined as 'Apoptotic'(middle right column); TO-PRO-3^{low} cells from non-A5^{high} population were defined as

‘Viable’(far right column). Axes of plots for each column and red arrows indicating gating steps are defined in top row for ‘Untreated DMSO’ sample.

Figure S2. Gating strategy and representative FACS dot plots utilised in Figure 1 C-D to assess cell death in THP-1 cells. Cells double-stained with TO-PRO-3 and A5-FITC were gated in four stages to identify ‘viable’, ‘apoptotic’ and ‘necrotic’ (membrane permeabilized) cells, as described for Figure S1.

Figure S3. Representative SyproRuby stained SDS-PAGE gel used in protein quantification. Representative SDS-PAGE gel demonstrating relative protein yields of whole cell lysate and EVs isolated via 2k, 16k and 100k centrifugation, as determined by SyproRuby staining. Data are representative of two independent experiments.

Figure S4. Representative protein quantification and fold increase in UV 24 h total protein yield vs UV 4h. (A) Representative quantification of total protein yields from Figure S3, as determined by densitometry analysis using ImageJ software. (B) Fold increase in UV 24 h protein yield above UV 4h protein yield, in which protein yield of UV 4h is normalised to a value of 1. Data are representative of two independent experiments. In (B), error bars represent St Dev.

Figure S5. Full-length blot image of EV marker Alix. Full, uncropped image of immunoblot membrane including molecular weight (MW) ladder from Figure 6 in which EV marker Alix is indicated by dashed boxes for (A) Untreated 4 h and UV 4 h, (B) LPS/nigericin and Hyperthermic stress and (C) Untreated 24 h and UV 24 h samples.

Figure S6. Full-length blot image of EV marker ARF6. Full, uncropped image of immunoblot membrane from Figure 6 in which EV marker ARF6 is indicated by dashed boxes for (A) Untreated 4 h and UV 4 h, (C) LPS/nigericin and Hyperthermic stress and (E) Untreated 24 h and UV 24 h samples. Additional images for each blot in which images are of poorer quality but MW ladders are present, can be seen in (B) Untreated 4 h and UV 4 h, (D) LPS/nigericin and Hyperthermic stress and (F) Untreated 24 h and UV 24 h.

Figure S7. Full-length blot image of EV marker Calreticulin. Full, uncropped image of immunoblot membrane including molecular weight (MW) ladder from Figure 6 in which EV marker Calreticulin is indicated by dashed boxes for (A) Untreated 4 h and UV 4 h, (B) LPS/nigericin and Hyperthermic stress and (C) Untreated 24 h and UV 24 h samples.

Figure S8. Full-length blot image of EV marker CD81. Full, uncropped image of immunoblot membrane including molecular weight (MW) ladder from Figure 6 in which EV marker CD81 is

indicated by dashed boxes for (A) Untreated 4 h and UV 4 h, (B) LPS/nigericin and Hyperthermic stress and (C) Untreated 24 h and UV 24 h samples.

Figure S9. Analysis of LDH release and EV generation by LPS/nigericin-treated THP-1 cells over extended times. (A) NTA analysis of supernatants of THP-1 cells subjected to LPS/nigericin treatment collected 90 min and 180 min, merged with Figure 3C timepoints (15-60 min). Supernatants were isolated for NTA analysis following 300 *g* and subsequent 2000 *g* centrifugation. Values displayed represent particle concentration above levels of untreated samples. (B) Total membrane lysis as determined by LDH cytotoxicity assay was performed at additional 90 min and 180 min timepoints and merged with Figure 3D timepoints. Additional 90 min and 180 min timepoints in Figure S9 are displayed in red. Data are representative of two independent experiments. Error bars = S.E.M. (N=3).

Hyperthermic stress	Size (d.nm)	% Intensity	St Dev (d.nm)
Peak 1	771.1	84.9	311.3
Peak 2	111.9	12.7	29.5
Peak 3	5216	2.4	465.9
**PdI**	**0.527**		

UV 24 h	Size (d.nm)	% Intensity	St Dev (d.nm)
Peak 1	1187	83.8	580.3
Peak 2	114.6	10.6	28.66
Peak 3	4799	5.6	711.5
**PdI**	**0.549**		

LPS + nigericin	Size (d.nm)	% Intensity	St Dev (d.nm)
Peak 1	727.4	86.2	268.1
Peak 2	65.19	11.6	21.66
Peak 3	5403	2.2	307.5
**PdI**	**0.534**		

UV 4 h	Size (d.nm)	% Intensity	St Dev (d.nm)
Peak 1	925.9	86.1	371.8
Peak 2	114.8	12.1	27.87
Peak 3	5194	1.7	478.1
**PdI**	**0.571**		

Supplementary Table 1. DLS modelling data.

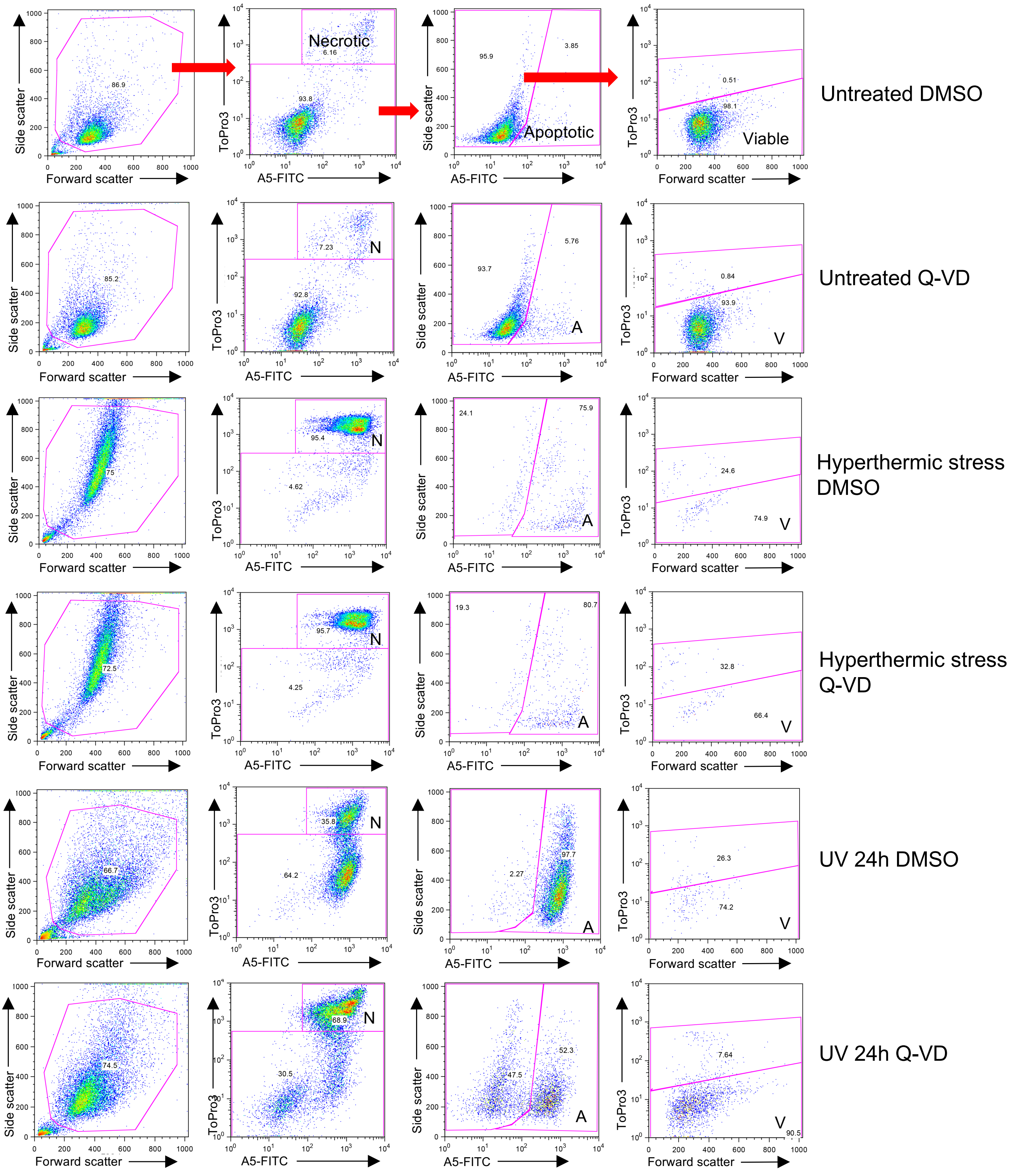

Supplementary Figure S1. FACS plots displaying gating strategy used in Figures 1A & 1B.

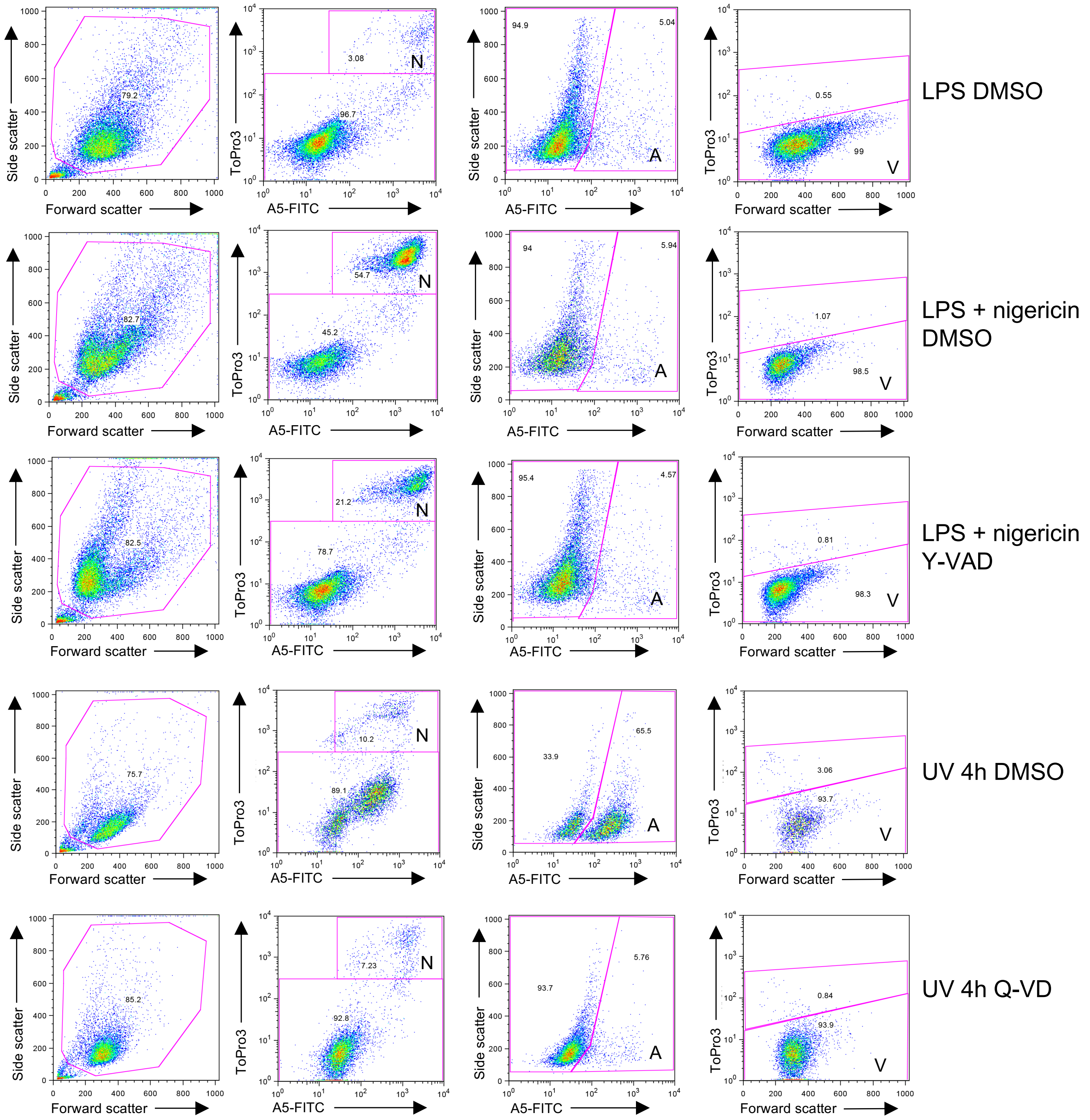

Supplementary Figure S2. FACS plots displaying gating strategy used in Figures 1C & 1D.

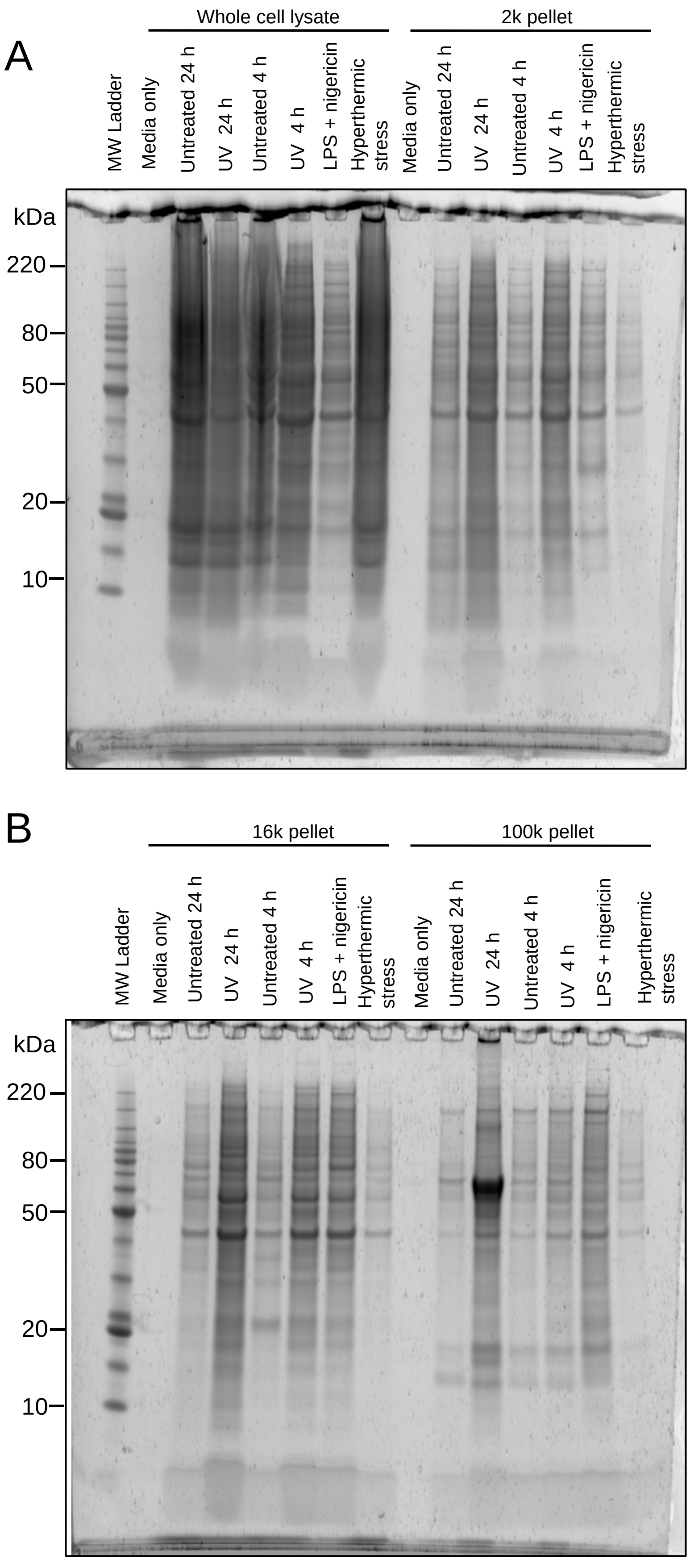

Figure S3. Representative SyproRuby stained SDS-PAGE gel used in protein quantification.

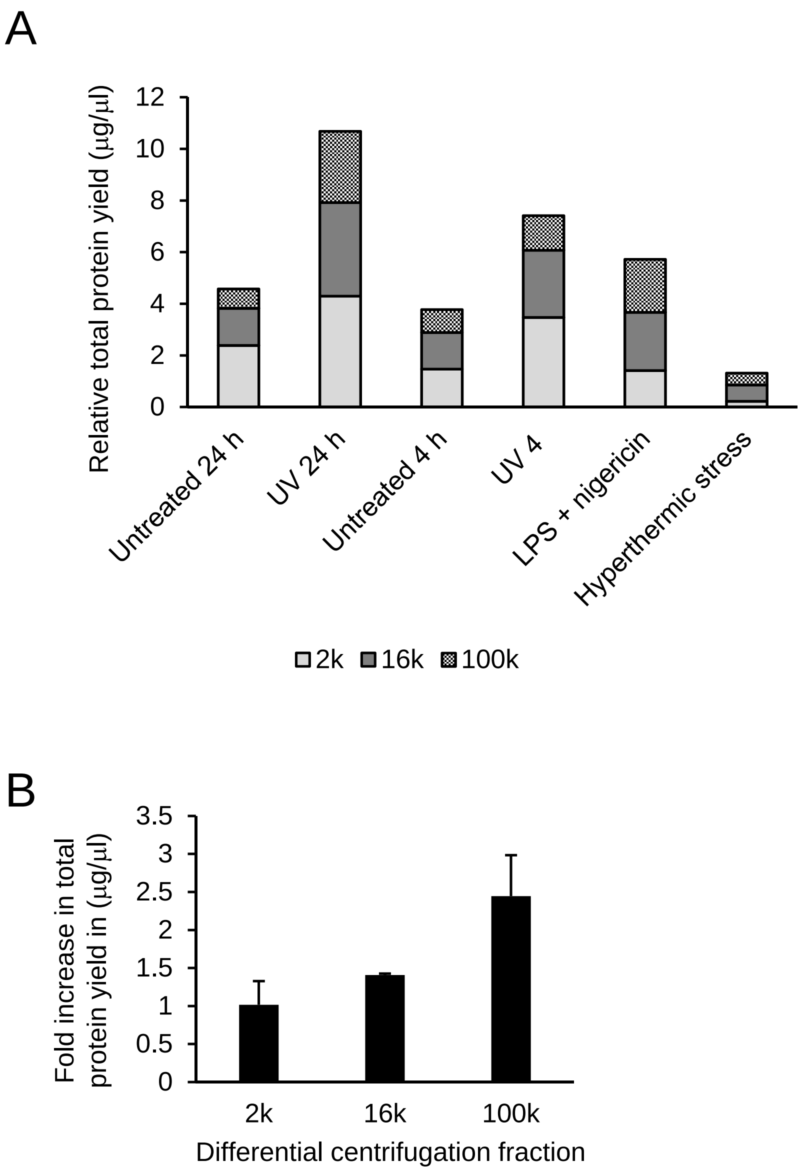

Figure S4. Representative protein quantification and fold increase in UV 24 h total protein yield vs UV 4h.

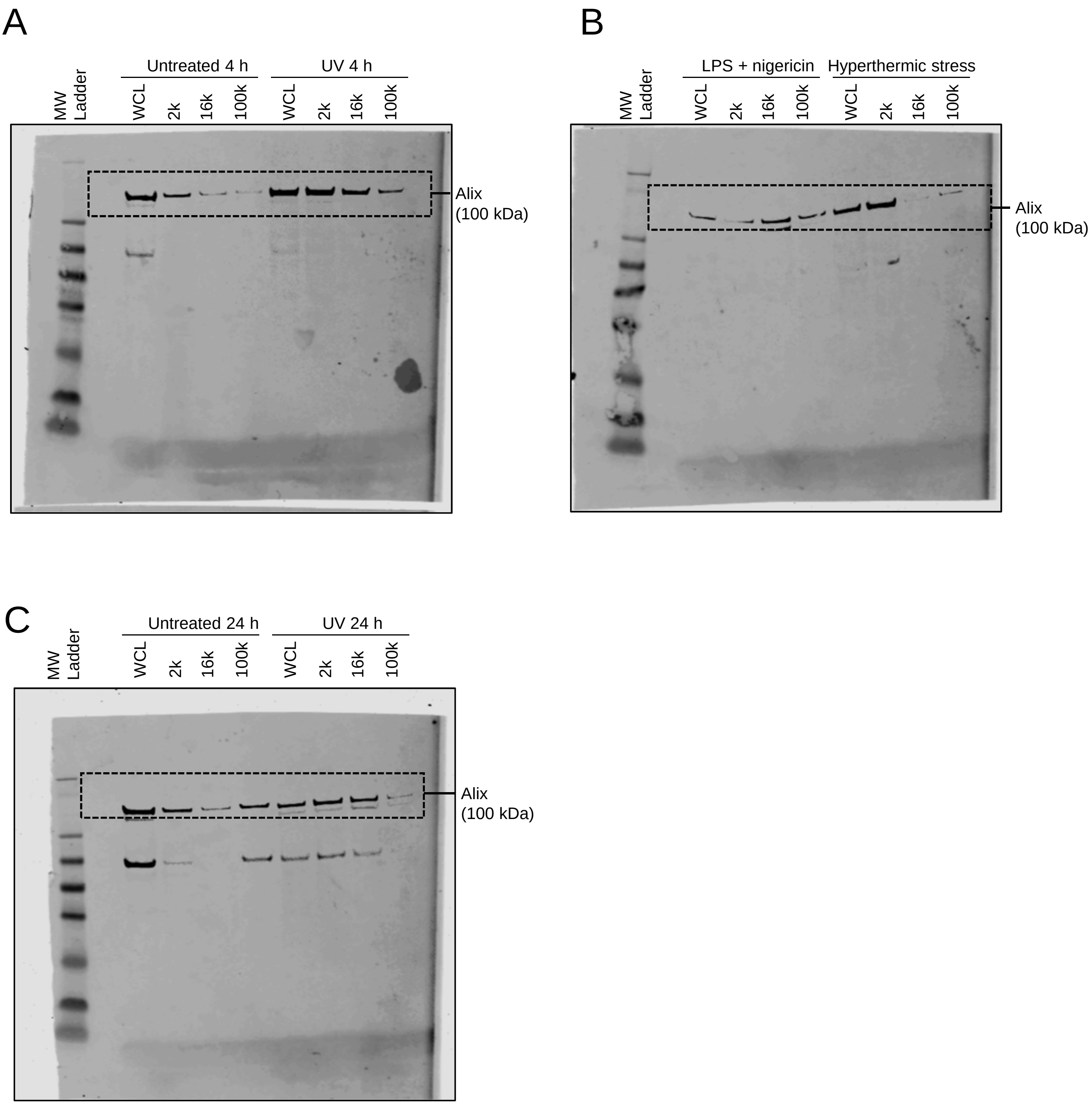

Figure S5. Full-length blot of Alix from Figure 6.

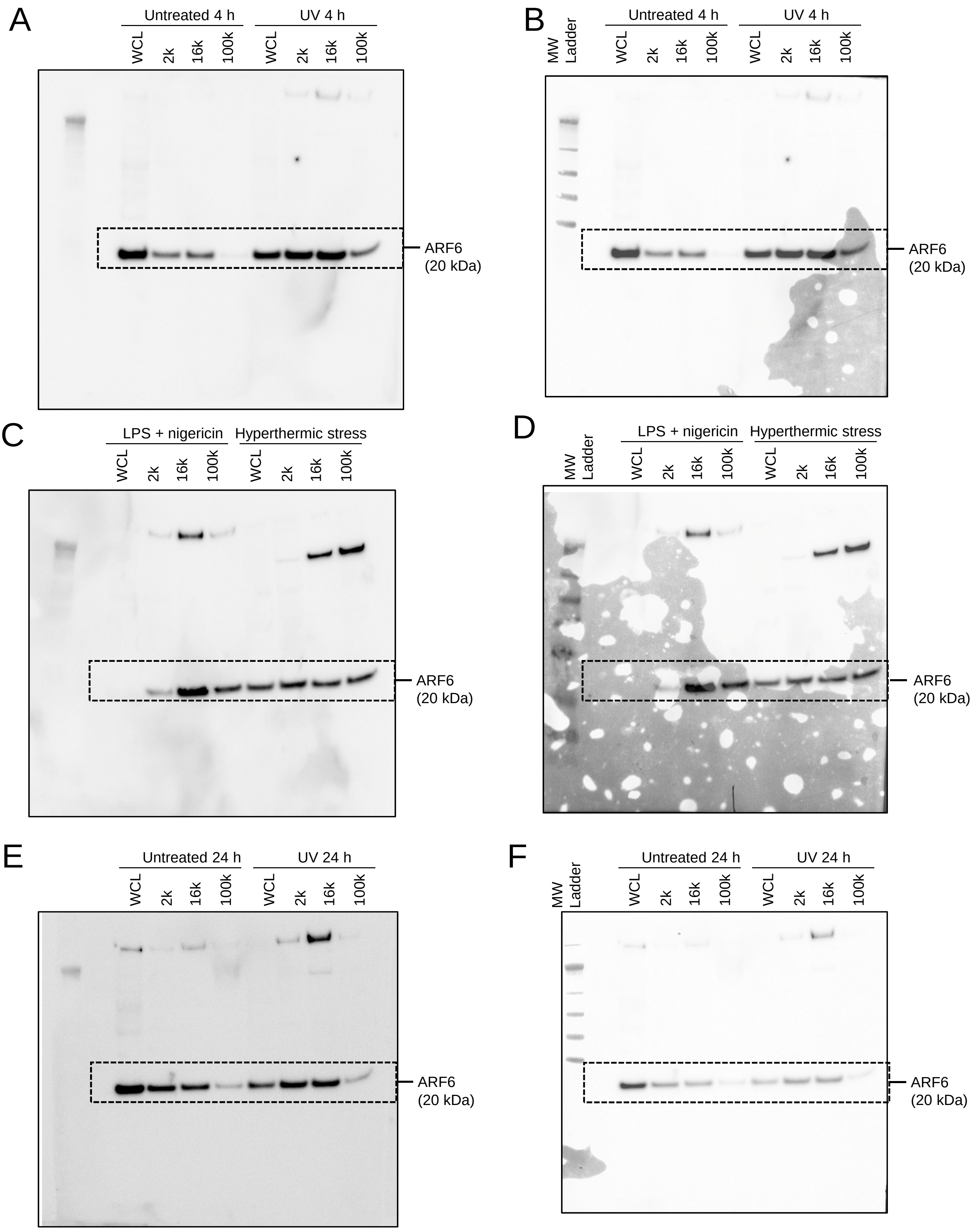

Figure S6. Full-length blot of ARF6 from Figure 6.

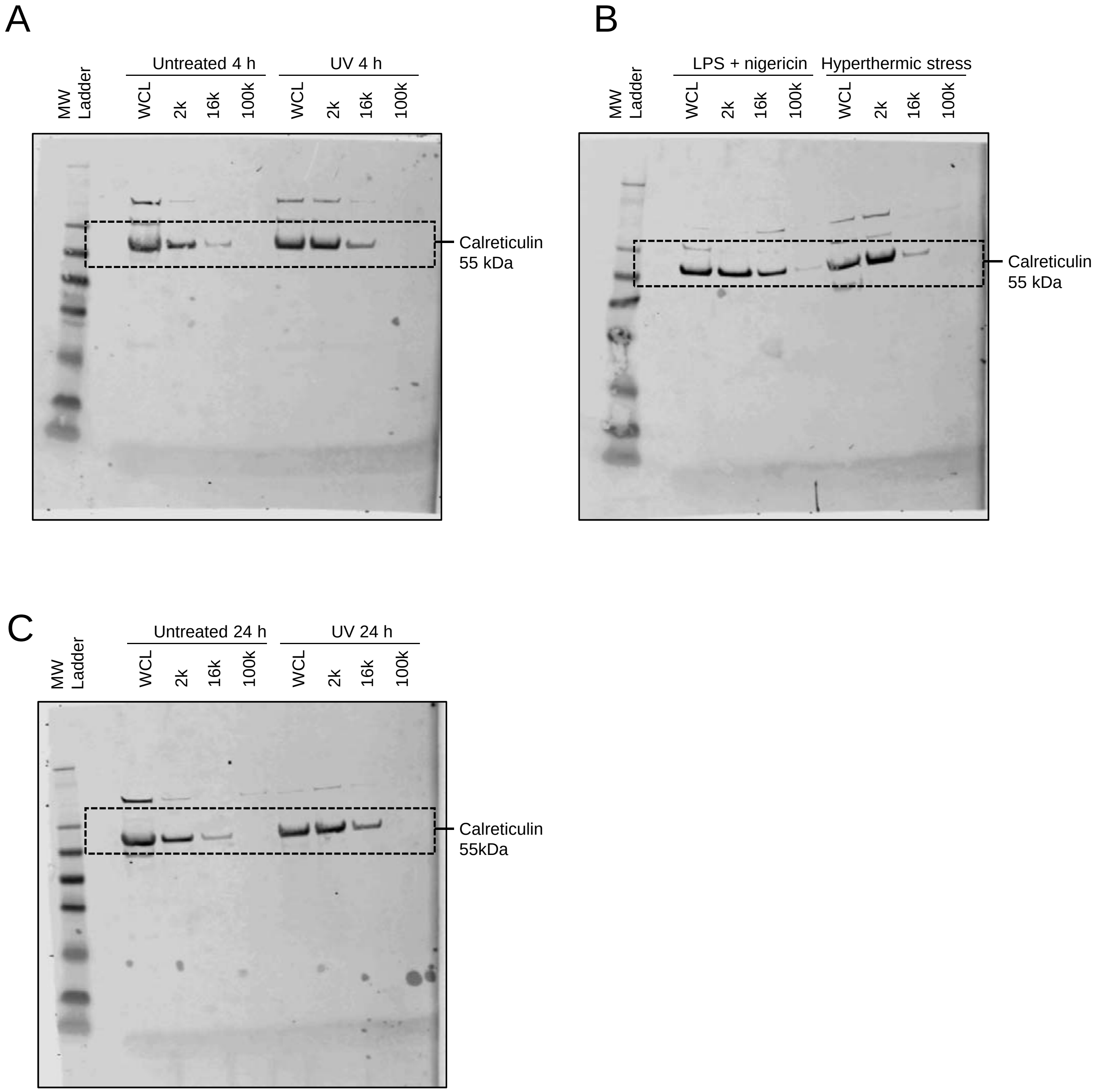

Figure S7. Full-length blot of Calreticulin from Figure 6.

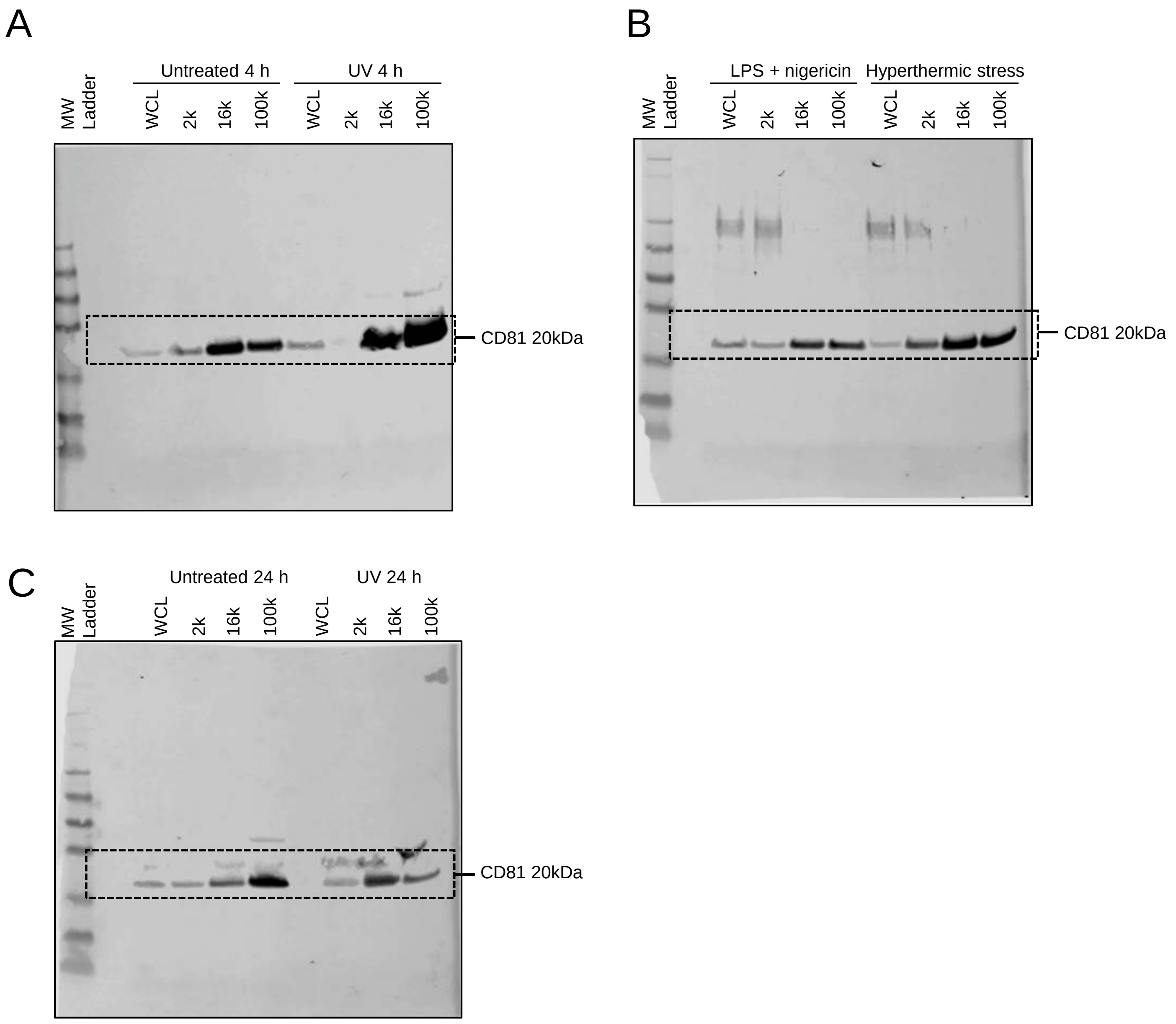

Figure S8. Full-length blot of CD81 from Figure 6.

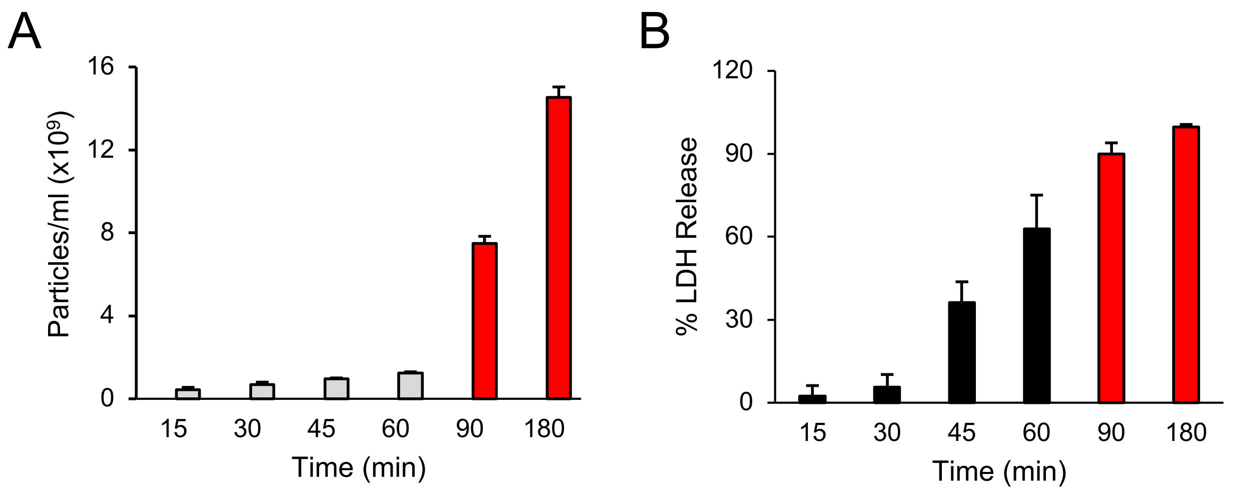

Figure S9. Analysis of LDH release and EV generation by LPS/nigericin-treated THP-1 cells over extended times.
